# Inanspruchnahme von Notaufnahmen durch sog. Mehrfachnutzer/-innen: Ergebnisse einer prospektiven Studie unter besonderer Berücksichtigung des Migrationsstatus

**DOI:** 10.1007/s10049-021-00851-7

**Published:** 2021-02-24

**Authors:** Matthias David, Rolf Richter, Baharan Naghavi, Theda Borde, Oliver Razum, Rajan Somasundaram, Hendrike Stein, Jalid Sehouli

**Affiliations:** 1 Campus Virchow-Klinikum, Klinik für Gynäkologie mit Zentrum für onkologische Chirurgie, Charité – Universitätsmedizin Berlin, Augustenburger Platz 1, 13353 Berlin, Deutschland; 2 Charité Comprehensive Cancer Center, Charité – Universitätsmedizin Berlin, Berlin, Deutschland; 3 Alice Salomon-Hochschule Berlin, Berlin, Deutschland; 4 Fakultät für Gesundheitswissenschaften, AG 3: Epidemiologie und International Public Health, Universität Bielefeld, Bielefeld, Deutschland; 5 Zentrale Notaufnahme, Campus Benjamin Franklin, Charité – Universitätsmedizin Berlin, Berlin, Deutschland; 6 Rettungsstelle, Vivantes Klinikum Berlin-Neukölln, Berlin, Deutschland

**Keywords:** Inanspruchnahme, Notfall, Notaufnahme, Migranten, Migrationshintergrund, Utilization, Emergency, Emergency room, Migrants, Migration background

## Abstract

**Fragestellung:**

Welche prädiktiven Faktoren lassen sich für die Gruppe der sog. Mehrfachnutzer (MFN; 4 und mehr Inanspruchnahmen einer Notaufnahme [NA] in den letzten 12 Monaten) finden? Sind Personen mit Migrationshintergrund häufiger in die Gruppe der MFN einzuordnen?

**Methodik:**

Konsekutive Patienten, die von Juli 2017 bis Juli 2018 drei Klinik-NA in Berlin aufsuchten. Mittels Fragebogen Erfassung von Erkrankungen, Gründen für den NA-Besuch und sozioökonomischen Faktoren. Die Unterschiede zwischen Migranten (1. Generation), ihren Nachkommen (2. Generation) und Nichtmigranten wurden mithilfe der logistischen Regression bewertet.

**Ergebnisse:**

2339 Patienten konnten in die Auswertung einbezogen werden (Rücklaufrate 56 %), davon hatten 901 einen Migrationshintergrund. Bei jungen Frauen (<30 Jahre), chronisch Kranken, Schwangeren, Patientinnen und Patienten mit starken Beschwerden und Personen mit (selbsteingeschätzter) mittlerer und schlechter „Gesundheitsqualität“ sowie solchen ohne ärztliche Zuweisung war die Chance für eine Mehrfachnutzung der NA größer.

**Schlussfolgerung:**

MFN belasten das ohnehin zunehmende Patientenvolumen von NA. Sie stellen jedoch eine heterogene Patientengruppe dar, unter der Menschen mit Migrationshintergrund nicht häufiger vertreten sind. Weitere Untersuchungen sind erforderlich, um die Faktoren, die zu einer häufigen Inanspruchnahme führen, besser zu verstehen und wirksame Strategien zu entwickeln, um den komplexen Gesundheitsbedürfnissen von MFN gerecht zu werden.

## Abstract (en)

**Objectives:**

Can we identify predictive factors for the group of so-called multiple users (MU; 4 and more uses of an emergency department [ED] in the past 12 months)? Are people with a migration background more likely to be classified in the MU group?

**Methodology:**

Included were consecutive patients who visited three EDs in Berlin from July 2017 to July 2018. Using a questionnaire, diseases, reasons for visiting the ED and socioeconomic factors were recorded. Comparisons between migrants (1st generation), their descendants (2nd generation) and nonmigrants were assessed using logistic regression.

**Results:**

A total of 2339 patients were included in the evaluation (repeat rate 56%), of which 901 had a migration background. Young women (<30 years), chronically ill, pregnant women, patients with severe complaints and people with (self-assessed) moderate and poor health quality as well as those without medical referral had a greater chance of multiple use of ED.

**Conclusion:**

MU burden the already increasing patient volume of ED. However, they represent a heterogeneous group of patients, among whom people with a migration background are not common. Further research is warranted to better understand the factors that lead to frequent use and to develop effective strategies to address the complex health needs of MUs.

## Einleitung

Die erhöhte Inanspruchnahme von Notaufnahmen (NA) und deren temporäre Überfüllung sind ein globales Problem [1–3], dies hat auch die aktuelle COVID-19-Pandemie erneut vor Augen geführt [4]. Eine besondere Gruppe von Patienten sind die sog. Viel- oder Mehrfachnutzer (MFN), die NA überproportional häufig aufsuchen und diese damit vor zusätzliche Herausforderungen stellen [5]. Meist werden in der Literatur 4 oder mehr NA-Besuche pro Jahr als Grenzwert benutzt [6, 7]. Die Identifizierung dieser MFN ist aus gesundheitspolitischen Gründen notwendig und aus Sicht der Versorgungsforschung interessant, da diese Patientengruppe erhebliche medizinische Ressourcen binden und verbrauchen kann [6]. Kanzaria et al. (2017) berichten, dass sog. Vielnutzer 4–8 % der NA-Patienten und 21–28 % aller NA-Besuche ausmachen [8]. MFN stellen sich in der Wahrnehmung des NA-Personals häufig mit nicht dringend behandlungsbedürftigen Beschwerden und damit einhergehend einem als inadäquat empfundenen Ressourcenverbrauch vor [9]. Allerdings zeigt eine Reihe meist retrospektiver Studien, dass MFN einen schlechteren Gesundheitszustand aufgrund chronischer Erkrankungen aufweisen, was zu der erhöhten Inanspruchnahme führen kann [10]. Der Terminus „unangemessene NA-Nutzung“ wird international unterschiedlich definiert. Bei der Interpretation von Studienergebnissen sind regionale bzw. nationale Unterschiede in den Gesundheitssystemen und die jeweiligen sozioökonomischen und kulturellen Verhältnisse zu berücksichtigen [1].

Studien zur überproportionalen NA-Nutzung haben verschiedene soziale Determinanten, darunter auch den Faktor Migrationshintergrund, untersucht. Nach den Ergebnissen einer 2012 publizierten norwegischen Studie suchen Migrantinnen und Migranten NA insgesamt seltener als Einheimische auf [11], während andere Studien mit unterschiedlicher Studienqualität und Stichprobengröße insgesamt über eine deutlich höhere NA-Inanspruchnahme durch Migrantinnen und Migranten im Vergleich zu Einheimischen in Norwegen, Dänemark, Italien und der Schweiz berichten [12–16]. Von Isik et al. (2020) wurden 335.457 NA-Besuche in der Türkei des Jahres 2018 ausgewertet. Sog. MFN machten 6,8 % der Gesamtpatientenpopulation, aber 22,9 % aller NA-Besuche aus. Der Anteil von Frauen, jungen Menschen und nichtversicherten Patienten (davon 50 % Immigranten und Flüchtlinge) war in dieser Gruppe größer als bei den „Normalnutzern“ [10]. Auch Studien in der Schweiz [17], Italien [18] und Norwegen [19] berichteten darüber, dass sog. Vielnutzer von NA häufiger Zuwanderer waren.

Eine systematische Charakterisierung der Gruppe von Vielnutzern ist notwendig und sinnvoll, um die tatsächlichen medizinischen und psychosozialen Bedürfnisse dieser Patientenklientel zu erkennen und eine Verringerung der durch die häufige Inanspruchnahme verursachten Belastungen von NA zu erreichen. Im Zuge einer prospektiven Studie erhobene Daten zur NA-Inanspruchnahme sollten dahingehend analysiert werden, ob Personen mit Migrationshintergrund häufiger in die Gruppe der sog. Mehrfachnutzer (MFN) einzuordnen sind als Personen ohne Migrationshintergrund oder -erfahrung und welche prädiktiven Faktoren sich für die Gruppe der MFN finden lassen.

## Methodik

### Fragebögen.

Der Leitfaden für die standardisierten Interviews konzentrierte sich auf folgende Themenbereiche (insgesamt 51 Items): Zugangswege in und Erwartungen an die NA, Schmerz- und Beschwerdewahrnehmung, Eigenhilfemaßnahmen, Sozialdaten, Fragen zur allgemeinen Lebenslage sowie zu Migration/Akkulturation. Das Fragebogenpaket enthielt überwiegend vorgegebene Antwortkategorien. Die Datenerhebung erfolgte durch 3 Study Nurses, 10 studentische Mitarbeiterinnen sowie eine wissenschaftliche Mitarbeiterin/Projektkoordinatorin.

Die Zuordnung eines Migrationshintergrunds erfolgte auf Basis der von den Patientinnen und Patienten angegebenen Muttersprache und des angegebenen eigenen bzw. des Geburtsorts der Eltern. Es wurde zwischen Personen mit eigener Migrationserfahrung (selbst im Ausland geboren = 1. Generation) und den direkten Nachkommen (selbst in Deutschland geboren, mindestens ein Elternteil im Ausland geboren, keine eigene Migrationserfahrung = 2. Generation) unterschieden. Beide Definitionen entsprechen den Festlegungen des Statistischen Bundesamts [20]. Um unter den Personen mit einer eigenen oder familienbezogenen Migrationserfahrung auch den Faktor Akkulturation analysieren zu können, wurde der Grad der Akkulturation mithilfe des Frankfurter Akkulturationsfragebogens (FRAKK; [21]) erhoben.

Das Teilkollektiv der MFN wurde entsprechend der Antwort auf die Frage „Wie oft waren Sie in den letzten 12 Monaten Patient/-in einer Notfallambulanz?“ gebildet. Unter Bezugnahme auf eine Reihe internationaler Publikationen erfolgte die Zuordnung zum MFN-Kollektiv in Anlehnung an Kuek et al. (2018), wenn die Befragten zusätzlich zur aktuellen Inanspruchnahme eine NA schon mindestens 3‑mal innerhalb der letzten 12 Monate vor der Befragung aufgesucht hatten [22].

Die standardisierten, fragebogenbasierten Interviews wurden stets vor dem Arztkontakt in den NA durchgeführt. Das validierte Fragebogenpaket lag neben Deutsch auch in englischer, türkischer, arabischer und russischer Sprache vor.

Parallel zur Patientenbefragung wurden an den drei Berliner Studienstandorten Charité, Campus Virchow-Klinikum (CVK Gyn.), Charité, Campus Benjamin Franklin (CBF), und Vivantes Klinikum Neukölln (VKN) die Daten der sog. Erste-Hilfe-Scheine aller Patientinnen und Patienten, die die NA aufgesucht und vom Studienteam befragt wurden, erfasst: administrative Daten, die ärztliche Dokumentation zur Anamnese, die aktuelle Beschwerdesituation sowie das diagnostische und therapeutische Vorgehen zu jeder/jedem in die Studie eingeschlossenen Patientin und Patienten. Fragebogen- und Erste-Hilfe-Schein-Daten wurden zusammengeführt und dann anonymisiert.

### Ein- und Ausschlusskriterien.

Es wurden nur volljährige, einwilligungsfähige Personen einbezogen, die die NA der jeweiligen Standorte zwischen 9 und 23 Uhr aufsuchten. Von der Befragung ausgeschlossen wurden Personen, die nicht ansprechbar waren (z. B. wegen akuter Lebensgefahr, Alkoholisierung oder weil sie unter Drogen standen), und solche, die wegen der Behandlungsdringlichkeit unmittelbar und unverzüglich vom NA-Personal behandelt werden mussten. Ebenfalls nicht nochmals befragt wurden Patientinnen und Patienten, die im Zuge einer vorangegangenen Vorstellung in einer der drei Klinik-NA bereits an der Studie teilgenommen hatten.

### Statistik.

Die statistische Auswertung erfolgte mit den Datenverarbeitungsprogrammen Microsoft Excel sowie IBM SPSS Statistics Version 24. Für die Identifizierung statistischer Unterschiede wurde der Pearson-Chi^2^-Test bzw. Kendalls tau b für ordinale Daten verwendet, wobei eine statistische Signifikanz ab einer Irrtumswahrscheinlichkeit von *p* < 0,05 angenommen wurde.

Die Zufriedenheit mit der Gesundheit und dem Leben insgesamt (Angaben auf einer 11-stufigen Likert-Skala von „ganz und gar unzufrieden“ [=0] bis „ganz und gar zufrieden“ [=10]) wurde als „Gesundheitsqualität“ zusammengefasst. Für kontinuierliche Variablen wie Alter, Beschwerdestärke, Dringlichkeit und „Gesundheitsqualität“ wurden mittels Receiver-operator-characteristics(ROC)-Kurven Trennwerte zur Diskriminierung von Mehrfachnutzung gegenüber keiner Mehrfachnutzung ermittelt. Im weiteren Verlauf wurden multivariate logistische Regressionsmodelle schrittweise mit *p*_in_ = 0,05 und *p*_out_ = 0,10 analysiert, um Einflussfaktoren auf die MFN zu ermitteln. Odds Ratios (OR), kontrolliert für alle anderen Einflussfaktoren mit entsprechendem 95 %-Vertrauensbereich, wurden berechnet.

### Ethikvotum und Datenschutz.

Für die Studie liegt ein positives Ethikvotum der Ethikkommission der Charité, Campus Virchow-Klinikum (Antragsnummer: EA2/102/17) vor. Die „Satzung der Charité zur Sicherung guter wissenschaftlicher Praxis“ und die Vorgaben des Berliner Datenschutzgesetzes wurden beachtet.

### Förderung.

Die Studie wurde gefördert durch den Gemeinsamen Bundesausschuss/Innovationsausschuss (Förderkennzeichen: 01VSF16038).

## Ergebnisse

Die Datenerhebungsphase erfolgte an den drei Studienstandorten zeitlich gestaffelt (CVK Gyn.: 18.07.2017–29.06.2018; CBF: 23.08.2017–12.07.2018; VKN: 04.10.2017–10.07.2018) täglich zwischen 9.00 und 23.00 Uhr. Insgesamt wurden 4176 Patientinnen und Patienten zur Teilnahme an der Studie eingeladen, wovon 2339 dem zustimmten (Responserate 56 %). Häufigste dokumentierte Ablehnungsgründe waren: Patient/-in fühlt sich nicht wohl (25 %), mangelnde Motivation zur Teilnahme (19 %), sprachliche Probleme (14 %), Ablehnung der Teilnahme durch eine Begleitperson (8 %). Die Einzelinterviews mit den Patientinnen und Patienten dauerten im Durchschnitt 20 ± 8 min. Aufgrund fehlender Angaben wurden nachträglich 12 Patientinnen und Patienten aus der Studie ausgeschlossen, sodass letztlich 2327 vollständige Datensätze für die Auswertung verwendet werden konnten. Bei 2271 der 2327 (97,6 %) Befragten lagen auch die dazugehörigen Angaben aus den Erste-Hilfe-Scheinen vollständig für die Datenanalyse vor.

Es wurden insgesamt mehr Frauen in die Studie einbezogen, da am Standort Charité, Campus Virchow-Klinikum, ausschließlich Patientinnen der gynäkologischen NA befragt werden konnten; insgesamt wurden 1600 (69 %) Frauen und 724 (31 %) Männer in die Studie eingeschlossen. 901 (39 %) Befragte hatten einen Migrationshintergrund.

Zielkollektiv dieser Analyse ist die Subgruppe der sog. MFN (insgesamt 4 und mehr Inanspruchnahmen einer NA in den letzten 12 Monaten entsprechend den Eigenangaben der Befragten). Dies waren 10,7 % der Patientinnen und Patienten (*n* = 250). Der Anteil von Frauen (11 %) und Männern (10,5 %) war ähnlich (*p* = 0,392). Auch der Anteil der MFN unter den Befragten, die die Frage „Kennen Sie außer dieser Notfallambulanz noch andere Stellen, die in Notfällen ärztliche Hilfe anbieten?“ bejahten (62,2 % vs. 60,6 %; *p* = 0,336), unterschied sich kaum. Bei der Differenzierung nach Migrationsstatus zeigten sich keine statistisch signifikanten Unterschiede (*p* = 0,121): 11,3 % MFN ohne Migrationshintergrund, 8,9 % MFN bei den Personen mit eigener Migrationserfahrung (1. Generation), 13,3 % MFN in der Gruppe der Patientinnen und Patienten mit Migrationshintergrund (2. Generation). Innerhalb des Gesamtteilkollektivs mit Migrationserfahrung bzw. -hintergrund hatten selbsteingeschätzte Deutschkenntnisse (Gegenüberstellung „gar nicht“ bis „wenig“ vs. „einigermaßen“ bis „sehr gut“) keinen Einfluss (*p* = 0,501). In der Gruppe der MFN waren signifikant mehr Patientinnen und Patienten mit einer mittleren Ausbildung (12,8 %) gegenüber denen mit niedrigem (10,6 %) bzw. hohem Bildungsniveau (8,2 %; *p* = 0,035). Chronisch Kranke und solche mit regelmäßiger Medikamenteneinnahme waren deutlich häufiger in der Gruppe der MFN (13,1 vs. 6,8 bzw. 13,3 vs. 7,0; jeweils *p* = 0,000). Seltener in der Gruppe der MFN zu finden waren solche Patientinnen und Patienten, die angaben, dass die Entscheidung/Empfehlung für das Aufsuchen der NA von einem Arzt gekommen sei und nicht von der Person selbst bzw. Familienangehörigen oder Freunden, also medizinischen Laien (29,9 vs. 41,1 %; *p* = 0,001 [Tab. 1]). Häufige Mehrfachnutzung war mit stärkeren Beschwerden (tau b = 0,06; *p* < 0,001) und schlechterer „Gesundheitsqualität“ (tau b = −0,15; *p* < 0,001) verbunden, nicht aber mit einer stärkeren Dringlichkeitseinschätzung seitens der Patienten (tau b = 0,01; *p* = 0,403).

Die logistische, schrittweise durchgeführte Regression ergab, dass die Parameter Migrationshintergrund, Geschlecht, selbsteingeschätzte Deutschkenntnisse, „etwas selbst unternommen vor dem Aufsuchen der Klinik“, Vorhandensein einer Hausapotheke und selbsteingeschätzte Behandlungsdringlichkeit ohne signifikanten Effekt waren. Die Parameter „mittlerer Ausbildungsgrad“ und „chronisch krank“ waren häufiger mit einer Mehrfachinanspruchnahme von NA (≥4 in 12 Monaten) verbunden, die Variablen „höheres Lebensalter“ und „Entscheidung zum Aufsuchen der Notfallambulanz durch einen Arzt“ mit einer geringeren Chance dafür.

Mittels ROC-Kurve wurde ein Trennwert für die Altersgruppen festgelegt: Bei den Frauen war dies ein Alter von ≥ 30 Jahren und bei den Männern von ≥ 65 Jahren. Diese Werte wurden für die multivariate schrittweise logistische Regression verwendet. Demnach waren Frauen unter 30 Jahren häufiger MFN. Der Einfluss der Diagnose „Schwangerschaftsstörungen“ wurde überprüft: Im Frauenkollektiv < 30 Jahren waren 16,5 % MFN, bei den Patientinnen ≥ 30 Jahre 11,1 %. Die logistische Regression unter Ausschluss der Diagnose „Schwangerschaftsstörung“ bestätigte, dass Frauen unter 30 Jahre häufiger zur Gruppe der MFN gehören. Die Angaben zur Zufriedenheit mit der Gesundheit und dem Leben insgesamt (Angaben auf einer 11-stufigen Likert-Skala von „ganz und gar unzufrieden“ [=0] bis „ganz und gar zufrieden“ [=10]) wurden zusammengefasst als „Gesundheitsqualität“ ebenso in die Regression wie die angegebene Beschwerdestärke einbezogen. Die Analyseergebnisse zeigt Tab. 2.

**Table 1:** 

Variable	MFN (*n* = 250)	Nicht-MFN (*n* = 2058)	*p*-Wert
*Lebensalter* (in Jahren, Median)	50	49	0,340
*Frauen*	70,0	68,9	0,772
*Männer*	30,0	31,1	
*Ausbildung in Deutschland*			0,007
Niedrig	18,9	19,3	
Mittel	56,6	47,0	
Hoch	24,5	33,6	
*Zurzeit berufstätig*	26,9	42,3	0,001
*Familienstand*			0,170
Ledig	30,8	27,0	
Verheiratet	45,6	50,1	
Geschieden	14,0	10,9	
Verwitwet	9,6	11,9	
*Migrationshintergrund*			0,121
Kein Migrationshintergrund	63,6	61,0	
Migrationshintergrund: 1. Generation	22,4	27,8	
2. Generation	14,0	11,2	
*Selbsteingeschätzte Deutschkenntnisse (nur Personen mit Migrationshintergrund)*			0,873
Gar nicht bis wenig	15,1	14,6	
Einigermaßen bis sehr gut	84,9	85,4	
*Chronisch krank*	77,6	62,5	<0,001
*Stationäre Aufnahme erfolgt oder empfohlen/angeboten, aber abgelehnt*	37,4	31,5	0,079
*Hauptbeschwerden, (bezogen auf den Tag der Befragung:) seit wann?*			0,021
Etwa eine Stunde	4,1	4,1	
Heute	17,1	18,6	
Gestern	17,1	13,3	
Einige Tagen	18,7	26,1	
Ca. eine Woche	6,5	9,1	
Seit mehr als einer Woche	36,9	28,9	
*Entscheid für Aufsuchen der Notfallambulanz*			0,001
Pat. selbst/Angehörige/Bekannte	70,1	58,9	
Haus‑/Fach‑/Notarzt	29,9	41,1	

*MFN* Mehrfachnutzer/-innen

**Table 2:** 

Prädiktor	*p*	OR	95 %-KI	
			Untere	Obere
*Geschlecht/Alter*	0,007	–	–	–
Frauen ≥ 30 Jahre	0,589	1,14	0,71	1,84
Frauen < 30 Jahren	**0,007**	**2,21**	**1,25**	**3,91**
Männer < 65 Jahren	0,163	1,49	0,85	2,62
*Ausbildung*	0,038	–	–	–
Niedrig	0,778	0,94	0,60	1,47
Mittel	**0,042**	**1,43**	**1,01**	**2,03**
*Chronisch krank*	**0,000**	**2,01**	**1,39**	**2,90**
*Entscheid durch Arzt*	**0,001**	**0,58**	**0,42**	**0,79**
*Schwanger*	**0,001**	**2,18**	**1,40**	**3,41**
*Starke Schmerzen/Beschwerden*	**0,008**	**1,66**	**1,14**	**2,41**
*„Gesundheitsqualität“*	0,000	–	–	–
0–10	**0,000**	**5,20**	**3,33**	**8,13**
11–15	**0,000**	**2,97**	**1,94**	**4,56**

*KI* Konfidenzintervall, *OR* Odds Ratio

## Diskussion

Die „richtige“ bzw. „adäquate“ Nutzung von klinischen NA ist ein komplexes Thema. Trotz zahlreicher Veröffentlichungen sind die Belege für die Wirksamkeit von Interventionen zur Reduzierung der NA-Inanspruchnahme nach wie vor unzureichend. Es ist z. B. unklar, für welche spezifische Zielgruppe welche Art von Intervention erfolgreich ist [23]. Die Patientengruppe der MFN sollte besser charakterisiert werden, um diejenigen zu erreichen, denen bedarfsorientierte Interventionen nützen würden [24]. Ziel der vorliegenden Studie war es u. a., prädiktive Faktoren für die häufige Nutzung von NA, definiert als mindestens 4 NA-Inanspruchnahmen in 12 Monaten, zu entdecken und wiederkehrende Merkmale bei den MFN-Patienten zu erkennen, nicht aber zu bewerten, ob es sich um eine Fehlinanspruchnahme handelt.

Die aufgestellte Eingangshypothese, dass ein Migrationshintergrund mit einer höheren Chance für eine NA-Mehrfachnutzung verbunden ist, wurde nicht bestätigt. Auch die (selbsteingeschätzten) Deutschkenntnisse, die Migrationsgeneration und das Ausmaß der Akkulturation innerhalb der Gruppe der Patientinnen und Patienten mit eigener oder familienbezogener Migrationserfahrung hatten keinen signifikanten Einfluss auf die häufige NA-Nutzung. Anders als bei Bodenmann et al. (2015) erhöhten auch andere Faktoren wie Entfernung des Wohnorts der Patienten zur NA, Transport mit einem Rettungswagen und selbsteingeschätzte Dringlichkeit der Beschwerden die Chancen für eine häufige Nutzung der NA nicht, ganz unabhängig vom Vorliegen eines Migrationsstatus [17].

Unsere Analysen zeigen zusammengefasst, dass bei jungen Frauen (<30 Jahren), chronisch Kranken, Schwangeren, Patientinnen und Patienten mit starken Beschwerden und Personen mit (selbsteingeschätzter) mittlerer und schlechter „Gesundheitsqualität“ sowie solchen ohne ärztliche Zuweisung die Chance für Mehrfachnutzung größer ist. Giannouchos et al. (2019) berichten im Ergebnis eines Literaturreviews, dass die MFN international 4–16 % aller NA-Benutzer, jedoch 14–47 % der NA-Besuche ausmachten [25]. Die Mehrheit der MFN waren junge Erwachsene oder Erwachsene mittleren Alters, Frauen sowie Personen mit niedrigem sozioökonomischem Status [25]. Auch Bodenmann et al. (2015) berichten aus der Schweiz, dass die MFN eher jünger waren [17]. Medizinische Komorbiditäten waren bei älteren MFN signifikant häufiger. Kuek et al. (2018) unterscheiden zwei MFN-Gruppen: jüngere Patienten mit sozialen Problemen und ältere Patienten mit mehreren Erkrankungen [22]. Betroli-Avella et al. (2017) fanden in dem von ihnen untersuchten Kollektiv der MFN mehr Personen mit chronischen und häufig schweren somatischen Erkrankungen [26]. Auch in unserem Berliner Kollektiv war „chronisch krank sein“ häufiger mit einer Mehrfachinanspruchnahme von NA (*mehr als *4 Besuche in 12 Monaten) verbunden. Bisherige Anstrengungen zur Lenkung der Patientinnen und Patienten in eine angemessene Versorgungsinstanz waren offenbar ohne Erfolg. Muche-Borowski et al. (2019) stellten im Ergebnis ihrer Patientenbefragung in norddeutschen NA fest, dass ambulante Strukturen der untersuchten Klientel chronisch Kranker bekannt waren und auch genutzt wurden [27]. Es ist daher zu prüfen, inwieweit die Versorgungsstrukturen und Kommunikationsprozesse den Bedürfnissen und Bedarfen der Patientinnen und Patienten entsprechen. Exadaktylos und Srivastava (2020) prognostizieren, dass fehlende oder nicht genutzte Grundversorgungsangebote und steigende Zahlen chronischer Erkrankungen in einer alternden Bevölkerung auch in Zukunft zu einem Anstieg des Gesamtvolumens und der ambulanten Notfallpatienten führen werden [28]. Nach ihrer Meinung spielt das Entlassungsmanagement in die ambulante Weiterbetreuung eine entscheidende Rolle, um ein sog. Entlassungsversagen, wozu auch das wiederholte Aufsuchen der NFA zählt, zu verhindern. Eine Reihe von Faktoren, zu denen neben einem niedrigen sozioökonomischen Status, einem Migrationshintergrund, dem höheren Lebensalter sowie Komorbiditäten auch chronische Erkrankungen gehören, begünstige eine qualitativ schlechte Entlassung. Um das Risiko eines „Entlassungsversagens“ zu verringern, werden, z. B. auch für chronisch Kranke, Entlassungsinstruktionen und Erklärungen, telefonische Nachkontrollen und eine gute Koordination der Weiterbetreuung empfohlen [28]. Wenn die Entscheidung, die NA aufzusuchen, nach Angaben der von uns befragten Patientinnen und Patienten von einem Arzt getroffen oder zumindest empfohlen wurde, senkte dies die Chance für eine Mehrfachnutzung. Auch Bertoli-Avella et al. (2017) fanden in ihrer in den Niederlanden durchgeführten Studie, dass im Vergleich zu Nicht-MFN die MFN signifikant seltener „Selbsteinweiser“ waren, seltener mit dem Krankenwagen ins Krankenhaus transportiert wurden und bei der Ankunft in der NA eher einen niedrigeren Dringlichkeitscode erhielten [26]. Dies steht nicht unbedingt im Widerspruch zu unseren Ergebnissen, dass Personen, die ihre Beschwerden als eher stark einschätzen, mit einer höheren MFN-Chance verbunden waren. Andererseits legt ein unlängst publiziertes Review der zwischen 1997 und 2019 veröffentlichten Arbeiten nahe, dass chronische Schmerzen mit einer Mehrfachinanspruchnahme assoziiert sind [29].

### Stärken und Schwächen der Studie.

Es handelt sich um eine interdisziplinär und interprofessionell durchgeführte prospektive Studie an einem großen und repräsentativen Patientenkollektiv. Damit erfolgte erstmals eine Analyse von MFN in Deutschland, die insbesondere Patientinnen und Patienten mit Migrationshintergrund angemessen berücksichtigt hat. Das Fragebogenpaket lag in fünf für die NA-Nutzung relevanten Sprachen vor. Eine Besonderheit ist die Zusammenführung der Interviewdaten mit den ärztlich-klinischen Angaben der sog. Erste-Hilfe-Scheine. Als Limitationen sind aufzuführen: 1. Die Non-Response-Rate von 44 %, darunter 14 % Patientinnen und Patienten, die wegen sprachlicher Probleme nicht teilnehmen konnten oder wollten; 2. mit den Erste Hilfe-Scheinen wurden medizinische Routinedokumente genutzt; 3. die tägliche Befragung fand aus organisatorischen Gründen nur von 9 bis 23 Uhr statt.

In Bezug auf praktische Konsequenzen aus den Untersuchungsergebnissen sei ergänzend auf die Datenauswertungen von Slankamenac et al. (2019) hingewiesen, die zwei MFN-Gruppen in ihrem Untersuchungskollektiv mit jeweils unterschiedlichen Risikofaktoren differenzieren konnten: a) sog. wiederholte MFN, die immer mit den gleichen Beschwerden bzw. Krankheitsbilden in die NA kamen, und b) sog. häufige MFN, die bei jedem NA-Besuch an unterschiedlichen Beschwerden litten. Die „wiederholten Nutzer“ besuchten die NA häufiger aufgrund von Symptomen chronisch-obstruktiver Lungenerkrankungen [30]. Bertoli-Avella et al. (2017) verweisen außerdem darauf, dass 2 % der MFN in ihrem Untersuchungskollektiv sog. serielle MFN waren, definiert als Mehrfachnutzung im Zeitraum von 3 oder mehr aufeinanderfolgenden Jahren [26].

Unsere Ergebnisse sollten andere Arbeitsgruppen motivieren, zu diesem relevanten Thema weitere Studien durchzuführen.

## Fazit für die Praxis

- Eine systematische Untersuchung der Gruppe von sog. Mehrfachnutzern ist notwendig, um die medizinischen und psychosozialen Bedürfnisse dieser Patienten zu erkennen und die Belastungen durch die häufige Inanspruchnahme von NA zu verringern.
- Unsere Analysen zeigen zusammengefasst, dass bei jungen Frauen (<30 Jahren), chronisch Kranken, Schwangeren, Patientinnen und Patienten mit starken Beschwerden und Personen mit (selbsteingeschätzter) mittlerer und schlechter Gesundheitsqualität sowie solchen ohne ärztliche Zuweisung die Chance für Mehrfachnutzung größer ist.
- Die aufgestellte Eingangshypothese, dass ein Migrationshintergrund mit einer höheren Chance für eine Mehrfachnutzung der NA verbunden ist, wurde nicht bestätigt.
- Auch die (selbsteingeschätzten) Deutschkenntnisse, die Migrationsgeneration und das Ausmaß der Akkulturation innerhalb der Gruppe der Patientinnen und Patienten mit eigener oder familienbezogener Migrationserfahrung hatten keinen signifikanten Einfluss auf die häufige NA-Nutzung.
- Weiteren Untersuchungen vorbehalten bleibt die Klärung der Frage, ob und wie häufig eine NA-Mehrfachnutzung mit einer inadäquaten NA-Inanspruchnahme verbunden ist.

## References

[1] Berchet C (2015). Emergency care services: trends, drivers and interventions to manage the demand.

[2] Ko M, Lee Y, Chen C, Chou P, Chu D (2015). Prevalence of and predictors for frequent utilization of emergency department: a population-based study. Medicine (Baltimore).

[3] Nambiar D, Stoové M, Dietze P (2017). Frequent emergency department presentations among people who inject drugs: a record linkage study. Int J Drug Policy.

[4] Christ N, Hiller M, Stuber P, Nickel CH, Bingisser R (2020) Die Rolle der Notfallambulanzen in der COVI-19-Pandemie. https://www.aerzteblatt.de/treffer?mode=s&wo=17&s=Notfallversorgung&typ=1&nid=111358. Zugegriffen: 12. Apr. 2020

[5] van Tiel S, Rood PP, Bertoli-Avella AM, Erasmus V, Haagsma J, van Beeck E (2015). Systematic review of frequent users of emergency departments in non-US hospitals: state of the art. Eur J Emerg Med.

[6] Birmingham LE, Cochran T, Frey JA, Stiffler KA, Wilber ST (2017). Emergency department use and barriers to wellness: a survey of emergency department frequent users. BMC Emerg Med.

[7] Norman C, Mello M, Choi B (2016). Identifying frequent users of an urban emergency medical service using descriptive statistics and regression analyses. West J Emerg Med.

[8] Kanzaria HK, Niedzwiecki MJ, Montoy JC, Raven MC, Hsia RY (2017). Persistent frequent emergency department use: core group exhibits extreme levels of use for more than a decade. Health Aff (Millwood).

[9] Acosta AM, Lima MA (2015). Frequent users of emergency services: associated factors and reasons for seeking care. Rev Lat Am Enfermagem.

[10] Işık CG, Tandoğan M, Şafak T, Çevik Y (2020). Retrospective analyses of the frequent emergency department users. Eurasian J Emerg Med.

[11] Sandvik H, Hunskaar S, Diaz E (2012). Immigrants’ use of emergency primary health care in Norway: a registry-based observational study. BMC Health Serv Res.

[12] Ruud SE, Aga R, Natvig B, Hjortdahl P (2015). Use of emergency care services by immigrants—a survey of walk-in patients who attended the Oslo Accident and Emergency Outpatient Clinic. BMC Emerg Med.

[13] Nielsen SS, Yazici S, Petersen SG, Blaakilde AL, Krasnik A (2012). Use of cross-border healthcare services among ethnic Danes, Turkish immigrants and Turkish descendants in Denmark: a combined survey and registry study. BMC Health Serv Res.

[14] Zinelli M, Musetti V, Comelli I, Lippi G, Cervellin G (2014). Emergency department utilization rates and modalities among immigrant population. Emerg Care J.

[15] De Luca G, Ponzo M, Andrés AR (2013). Health care utilization by immigrants in Italy. Int J Health Care Finance Econ.

[16] Braun CT, Gnägi CR, Klingberg K, Srivastava D, Ricklin ME, Exadaktylos AK (2017). Migrationsspezifische Aspekte des Patientengutes einer großen europäischen universitären Notaufnahme über einen Zeitraum von zehn Jahren. Praxis.

[17] Bodenmann P, Baggio S, Iglesias K, Althaus F, Velonaki VS (2015). Characterizing the vulnerability of frequent emergency department users by applying a conceptual framework: a controlled, crosssectional study. Int J Equity Health.

[18] Spinogatti F, Civenti G, Conti V (2015). Ethnic differences in the utilization of mental health services in Lombardy (Italy): an epidemiological analysis. Soc Psychiatry Psychiatr Epidemiol.

[19] Diaz E, Gimeno-Feliu LA, Calderón-Larrañaga A, Prados-Torres A (2014). Frequent attenders in general practice and immigrant status in Norway: a nationwide cross-sectional study. Scand J Prim Health Care.

[20] Statistisches Bundesamt (2019) Bevölkerung und Erwerbstätigkeit. Bevölkerung mit Migrationshintergrund – Ergebnisse des Mikrozensus 2018 – Fachserie 1 Reihe 2.2. https://www.destatis.de/DE/Themen/Gesellschaft-Umwelt/Bevoelkerung/Migration-Integration/Publikationen/Downloads-Migration/migrationshintergrund-2010220187004.pdf?__blob=publicationFile. Zugegriffen: 20. Apr. 2020

[21] Bongard S, Pogge SF, Arslaner H, Rohrmann S, Hodapp V (2002). Acculturation and cardiovascular reactivity of second-generation Turkish migrants in Germany. J Psychosom Res.

[22] Kuek BJW, Li H, Yap S, Ng MXR, Ng YY, White AE, Ong MEH (2018). Characteristics of frequent users of emergency medical services in Singapore. Prehosp Emerg Care.

[23] Van den Heede K, Van de Voorde C (2016). Interventions to reduce emergency department utilisation: a review of reviews. Health Policy.

[24] Krieg C, Hudon C, Chouinard MC, Dufour I (2016). Indivdual predictors of frequent emergency department use: a scoping review. BMC Health Serv Res.

[25] Giannouchos TV, Kum HC, Foster MJ, Ohsfeldt RJ (2019). Characteristics and predictors of adult frequent emergency department users in the United States: a systematic literature review. J Eval Clin Pract.

[26] Bertoli-Avella AM, Haagsma JA, Van Tiel S, Erasmus V, Polinder S, Van Beeck E (2017). Frequent users of the emergency department services in the largest academic hospital in the Netherlands: a 5-year report. Eur J Emerg Med.

[27] Muche-Borowski C, Boczor S, Schäfer I, Kazek A, Hansen H, Oltrogge J, Giese S, Lühmann D, Scherer M (2019). Chronisch Kranke in deutschen Notaufnahmen. Querschnittsanalysen zu Konsultationsanlässen, Gründen für die Inanspruchnahme und Entlassungsdiagnosen. Bundesgesundheitsblatt Gesundheitsforschung Gesundheitsschutz.

[28] Exadaktylos A, Srivastava D (2020). Ambulante Entlassung aus der Notfallstation. Notfall Rettungsmed.

[29] Dépelteau A, Racine-Hemmings F, Lagueux É, Hudon C (2019). Chronic pain and frequent use of emergency department: a systematic review. Am J Emerg Med.

[30] Slankamenac K, Zehnder M, Tim O, Langner TO, Krähenmann K, Keller DI (2019). Recurrent emergency department users. J Clin Med.

